# Prevalence of dental caries and its association with body mass index among school-age children in Shenzhen, China

**DOI:** 10.1186/s12903-019-0950-y

**Published:** 2019-12-04

**Authors:** Yi-hong Cheng, Yi Liao, Ding-yan Chen, Yun Wang, Yu Wu

**Affiliations:** 1 Wuhan University, School of Stomatology, Wuhan, China; 2 Shenzhen Center for Disease Control and Prevention, Shenzhen, China

## Abstract

**Background:**

Dental caries and overweight/obesity are health problems with shared risk factors, but the relationships between caries and BMI need to be further explored. The objective was to evaluate the current status of dental caries and the association between dental caries and Body Mass Index (BMI) among primary and secondary school students in Shenzhen, China, during the 2016–2017 academic year.

**Methods:**

A population-based, cross-sectional study that enrolled a total of 1,196,004 students was conducted in Shenzhen. Physical and dental examinations were given to all primary and secondary school students by certified physicians and dentists following the national specification for student health examinations, and dental caries was diagnosed using WHO criteria. Descriptive analysis was applied to assess current oral health status and a multifactorial, logistic regression model was employed to evaluate the relationship between dental caries and obesity.

**Results:**

A total of 1,196,004 students participated in the census. Mean age of the participants was 10.3 years, ranging from 6 to 20 years. The prevalence of dental caries was 41.15% in the present study, which was higher among girls (42.88%) than that in boys (39.77%) with a *p*-value of < 0.001. Students in public schools showed a significantly lower (*p* < 0.001) caries prevalence (37.36%) compared with those in private schools (47.96%). The caries restoration rate of students in Shenzhen was only 10.30%, which means only one out of ten students with caries received restorations. The mean dmft and DMFT scores were 0.97 and 0.33, respectively. More girls (10.96%) had their teeth filled than boys (9.78%). The restoration rate was higher (*p* < 0.001) in public schools (11.73%) than in private ones (8.35%). Children who were overweight or obese had a lower risk of experiencing caries compared to those who were within a normal weight (OR = 0.74/0.64). Caries was inversely associated with BMI among primary and secondary school students in Shenzhen.

**Conclusions:**

The prevalence of dental caries among primary and secondary school children was found to be related to sex, type of schools, region, and BMI. Further studies and more government support are required to confirm the findings of this study and to address current oral health problems.

## Introduction

Dental caries is one of the most prevalent chronic diseases among children [1]. According to the China’s Fourth National Oral Health Epidemiological Survey of 2017 [2, 3], 70.1% of 5-year-old children had primary dental caries and 34.5% of 12-year-old students had experienced dental caries in their permanent teeth [4]. Previous surveys conducted in other locations in China [5–7] have indicated high caries rates and low restorative rates among primary and secondary school students. Unhealthy diet, such as high calorie food, has been reported as a significant determinant of the increased prevalence of dental caries [8]. In addition, dietary factors have also contributed to the rising prevalence of childhood overweight/obesity worldwide in past decades [9, 10]. Given the shared risk factors between dental caries and overweight/obesity in childhood, studies examining the relationship between these two health problems have been conducted. However, results of previous studies have so far been inconsistent. Some authors have found a positive association [11, 12], while others reported no relationship [13, 14] or a negative one [15].

Moreover, few studies on estimating current dental caries status and the relationship between caries and BMI have been conducted in Shenzhen. Shenzhen is the largest immigrant city in China, with immigrants accounting for more than 70% of the population [16]. Due to diversified customs and rapid economic growth, the lifestyle of Shenzhen’s population has changed dramatically. Given that dental caries, which can affect the growth and development of school-age children [17], and is largely preventable, an updated study on evaluating the current status and identifying associated factors in Shenzhen is warranted.

Therefore, in order to estimate the current status, to compare potential disparities between regions of dental caries, and to determinate whether there is any association between dental caries and BMI among primary and secondary school children in Shenzhen, a population-based survey of students’ health was conducted. Our results should increase the understanding of dental health in Shenzhen school children and provides scientific evidence for policy and legislation development.

## Methods

### Sample enrollment

During the 2016–2017 academic year, a population-based census of students’ health and corporeity was conducted among all the primary and secondary school students by the Shenzhen government. This census covered both public and private schools, including a total of 1,196,004 students.

### Data collection

Dental and physical examinations were given to students by physicians and dentists trained with the national criteria (Technical Specification for Student Health Examination, GB-T-26343 2010 criteria) [18]. A flat mouth mirror and CPI probe were used for the dental examination. One-to-one dental caries detection was performed for each child, and gingivitis, tartar, and periodontal depth were recorded. Caries was diagnosed according to WHO criteria and caries experience was measured as the DMFT (number of decayed, missing, and filled permanent teeth) while the dmft was used for primary teeth. The presence of caries was diagnosed if dmft/DMFT> 0.

BMI, as used in this study, was calculated as weight (kg) divided by squared height (m^2^). We applied cut-off criteria recommended by the Group of China Obesity Task Force and used a population-based reference curve obtained from the 2000 national physical fitness and health surveillance data [19]. Subjects were categorized as moderate/severe malnutrition, mild malnutrition, normal weight, overweight, and obese according to cutoff values stratified by sex and age [19]. Overweight was defined as between 85 and 95% on the reference curve, and obese as higher or equivalent to 95%.

Demographic information (age, sex, residence, type of school) was obtained from the Shenzhen students’ health surveillance system. This study used secondary data collected from students’ health census in Shenzhen. All the data has been anonymised, therefore ethical approval was not required to our study.

### Statistical analysis

Qualitative data is presented as frequency and proportion. The prevalence of dental caries, mean dmft/DMFT scores, and caries restoration rates were mapped according to districts. Mean dmft/DMFT is the average score of caries status in both primary and permanent teeth, and caries restoration rate is the proportion of students who received restoration treatment among students with caries. Chi-square tests were conducted to compare the differences in caries prevalence and restoration rates by socioeconomic groups, such as age and type of school. Analysis of Variance (ANOVA) was applied to compare the mean dmft/DMFT scores by socioeconomic groups. A Multifactorial logistical regression model was applied to estimate the association between dental caries prevalence and BMI. District, sex, age group, and type of school were adjusted in this model. A *p*-value of less than 0.05 was considered statistically significant. Statistical analysis was performed using SAS 9.2 software.

## Results

A total of 1,196,004 students, including 665,728 boys (55.66%) and 530,276 girls (44.34%) participated in this survey. The overall prevalence of dental caries was 41.15% (Table 1), with a higher prevalence among girls than boys (*p* < 0.01). Age was significantly inversely associated with caries. Average restoration rates in these students remained relatively low (10.30%) compared with the high caries prevalence.

**Table 1:** Number of participants and their dental caries status stratified by age, types of school, and sex

	N			Prevalence of dental caries (%)					Mean dmft/DMFT score					Caries restoration rates (%)				
	Boys	Girls	Total	Boys	Girls	Total	*χ*^*2*^	*P* value	Boys	Girls	Total	F/t value	*P* value	Boys	Girls	Total	*χ*^*2*^	*p*-value
Age (Years)																		
6	38,338	32,401	70,739	52.62	54.15	53.32			1.98	2.02	2.00			7.51	7.58	7.54		
7	88,386	71,384	159,770	55.12	57.02	55.97			2.00	2.05	2.02			8.80	8.95	8.87		
8	90,110	72,901	163,011	57.82	59.62	58.62			1.98	2.02	2.00			9.22	9.57	9.38		
9	84,992	68,487	153,479	53.55	54.26	53.87			1.65	1.62	1.64			9.09	9.67	9.35		
10	75,091	58,468	133,559	44.89	44.57	44.75			1.18	1.12	1.15			8.95	9.92	9.37		
11	64,922	50,215	115,137	32.02	33.74	32.77			0.72	0.75	0.73			10.70	11.52	11.06		
12	60,599	47,100	107,699	21.80	25.87	23.58			0.43	0.54	0.48			12.55	13.31	12.88		
13	48,211	37,106	85,317	19.32	25.94	22.20			0.38	0.55	0.45			13.62	15.64	14.50		
14	42,200	32,570	74,770	19.52	26.64	22.62			0.40	0.59	0.48			16.18	20.87	18.22		
15	31,220	25,328	56,548	18.57	25.69	21.76			0.38	0.57	0.47			20.01	24.21	21.89		
16	26,237	22,276	48,513	17.21	24.93	20.75			0.34	0.55	0.44			25.32	33.34	29.00		
17	12,198	9692	21,890	17.36	24.58	20.56			0.36	0.56	0.45			28.49	35.23	31.47		
18+	3224	2348	5572	17.25	24.02	20.10	112,617.0	< 0.001	0.35	0.53	0.43	11,346.9	< 0.001	24.98	25.17	25.06	15,876.6	< 0.001
BMI																		
Moderate malnutrition	29,545	19,165	48,710	50.08	51.55	50.66			1.65	1.65	1.65			9.21	10.49	9.72		
Mild malnutrition	38,648	24,896	63,544	42.89	51.84	46.40			1.30	1.63	1.44			10.34	10.24	10.30		
Normal	425,694	406,238	831,932	41.61	43.17	42.37			1.28	1.28	1.28			9.66	10.98	10.31		
Overweight	95,514	46,360	141,874	33.16	37.77	34.66			0.94	1.09	0.99			10.29	11.85	10.85		
Obese	68,857	28,784	97,641	33.21	37.27	34.41	7326.9	< 0.001	0.95	1.06	0.98	1576.8	< 0.001	10.08	10.49	10.21	55.4	< 0.001
Type of school																		
Public	424,856	344,130	768,986	36.04	39.00	37.36			1.09	1.14	1.11			10.86	12.81	11.73		
Private	240,872	186,146	427,018	46.35	50.05	47.96	12,740.5	< 0.001	1.43	1.53	1.47	−88.9	< 0.001	8.32	8.38	8.35	3283.6	< 0.001
Total	665,728	530,276	1,196,004	39.77	42.88	41.15			1.21	1.28	1.24			9.78	10.96	10.30		

**Fig. 1:**
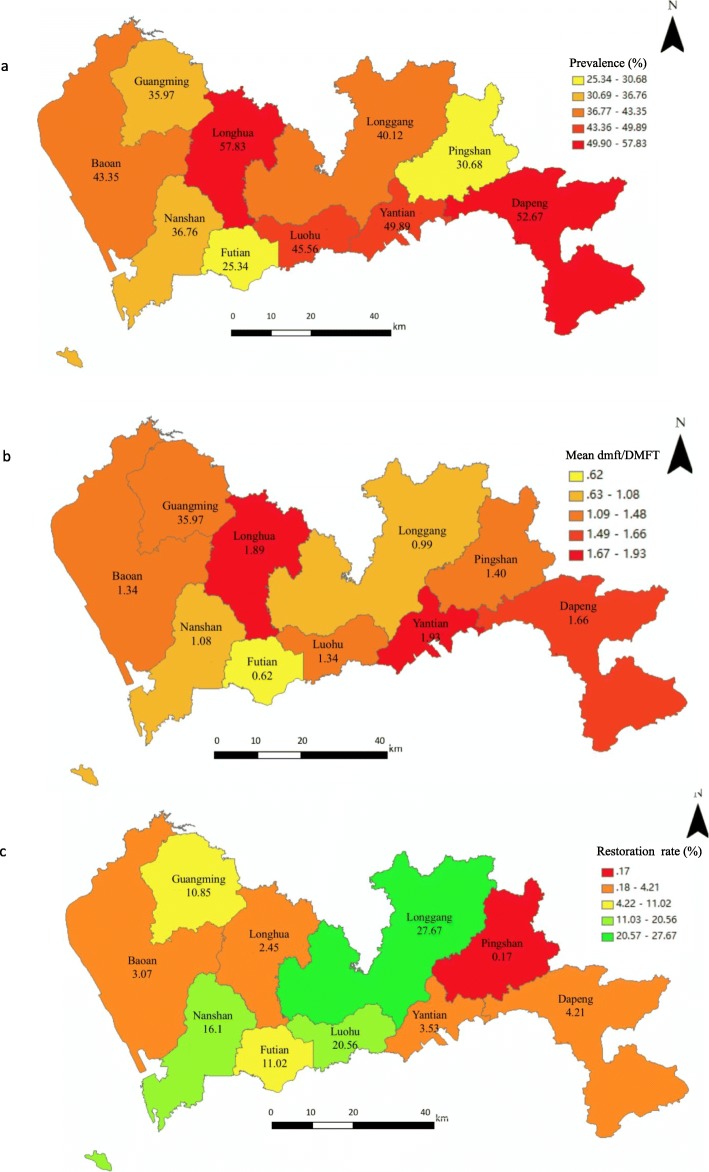
Maps of prevalence, mean dmft/DMFT score, and caries restoration rates by region

Dental caries status has been explored by administrative districts and types of school (Fig. 1, Table 1). Significant differences in caries status were found across districts (*p* < 0.001). The Longhua district showed the highest caries prevalence (57.83%), followed by the Dapeng new district (52.76%). The lowest caries prevalence and mean dmft/DMFT score were found in the Futian district. The Longgang district showed the highest restoration rates (27.67%), while only 0.17% of children with caries in the Pingshan district had their cavities restored. Students from public schools showed lower prevalence of caries and mean dmft/DMFT scores as well as higher caries restoration rates than those in private schools (Table 1).

As listed in Table 2, the overall prevalence of primary teeth with caries was 27.14%, with a higher prevalence among girls (27.32%) than boys (26.99%). With increasing age, the prevalence of caries in primary teeth decreased and the prevalence of caries in permanent teeth increased. The highest caries restoration rate was observed in the 12-year-old age group (17.24%) for primary teeth and in the 17-year-old age group (33.32%) for permanent teeth.

**Table 2:** Dental caries status of primary teeth and permanent teeth

Variables	N	Primary teeth			Permanent teeth		
		Prevalence of dental caries (%)	Mean dmft score	Caries restoration rates (%)	Prevalence of dental caries (%)	Mean DMFT score	Caries restoration rates (%)
Sex							
Boys	665,728	26.99	0.97	9.67	12.17	0.29	10.14
Girls	530,276	27.32	0.96	10.08	15.44	0.37	13.23
Age (Year)							
6	70,739	45.97	1.92	8.08	6.18	0.20	2.41
7	159,770	48.07	1.91	9.29	6.85	0.20	4.51
8	163,011	49.13	1.83	9.76	9.56	0.25	6.50
9	153,479	42.44	1.39	10.14	12.49	0.31	5.68
10	133,559	30.13	0.84	10.69	15.40	0.36	6.22
11	115,137	16.41	0.39	13.75	16.59	0.37	8.22
12	107,699	6.40	0.13	17.24	16.34	0.36	11.35
13	85,317	2.18	0.04	15.29	18.58	0.42	14.63
14	74,770	1.21	0.02	12.03	19.81	0.46	19.02
15	56,548	1.00	0.02	9.37	19.17	0.45	22.92
16	48,513	0.92	0.02	6.63	18.57	0.42	30.90
17	21,890	0.82	0.02	3.99	18.47	0.44	33.32
18+	5572	0.34	0.02	2.11	17.98	0.41	26.01
Total	1,196,004	27.14	0.97	9.85	13.62	0.33	11.72

**Fig. 2:**
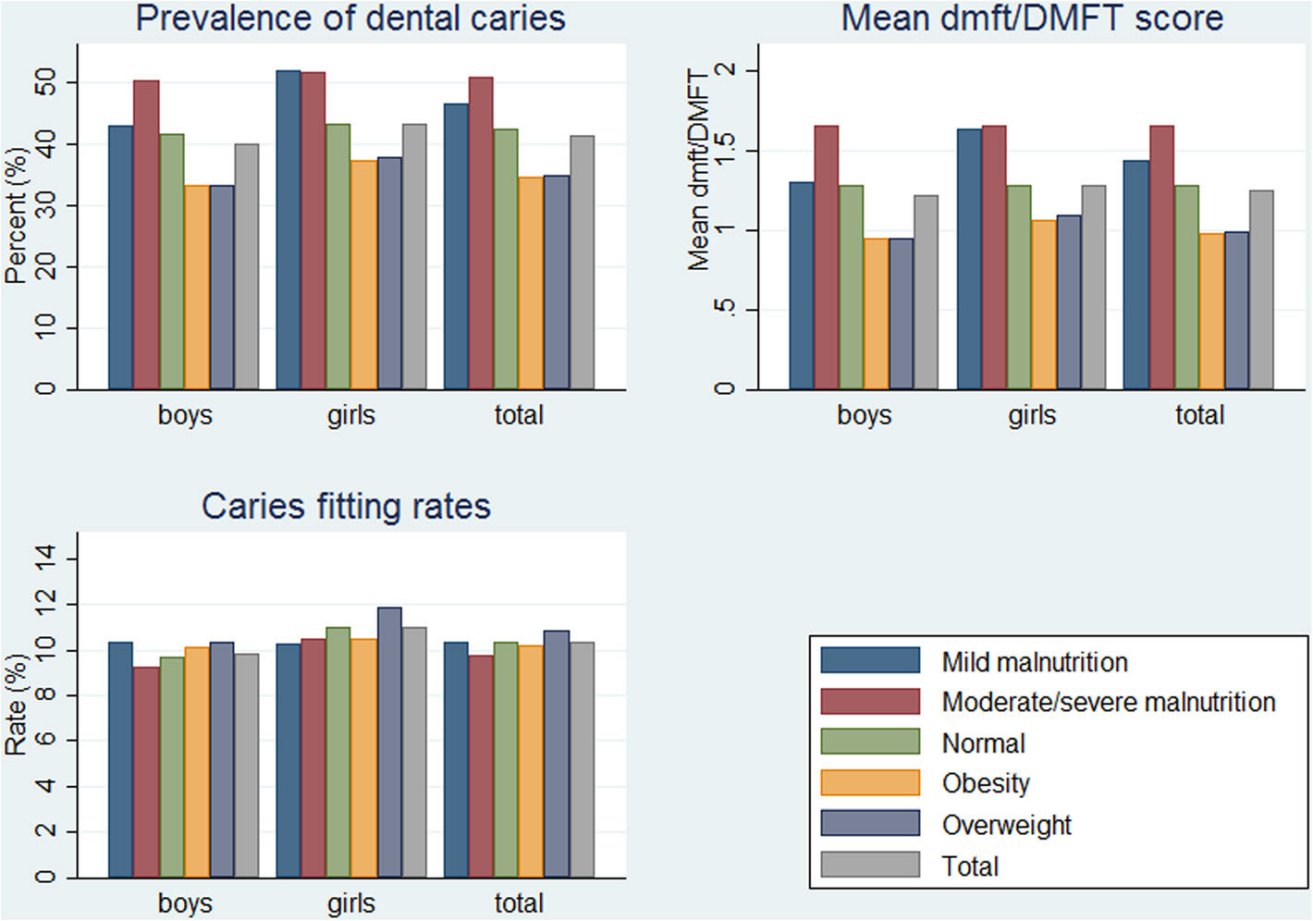
Dental caries status in each BMI groups based on Chinese criteria

According to the results of multifactorial logistical regression model (Table 3), the prevalence of dental caries among primary and secondary school children was found to be related to sex, type of schools, region, and BMI. Boys were less likely to have caries than girls (OR = 0.90), and students from private school had higher risk of experiencing dental caries compared to those from public school (OR = 1.23). Based on BMI criteria in China, 70.28% of students were categorized as having normal weight, and about 20.23% were overweight or obese. The association between caries and BMI is demonstrated in Table 3, after adjusting the other demographic variables. Caries were more likely observed among children with malnutrition, and the difference was statistically significant (*p* < 0.001). Children who were overweight or obese had a lower risk of experiencing caries compared to those who were within a normal weight (OR = 0.74/0.64), while students with moderate/mild malnutrition had a higher risk (1.19/1.17). Also, there was a significantly higher mean dmft/DMFT score among students with low BMI compared with those who were overweight or obese (p < 0.001) (Fig. 2). No significant difference in caries restoration rates was observed across BMI groups.

**Table 3:** Results of multifactorial logistical regression model

Variables	OR	95% CI		*P*
BMI				
Normal	1			
Moderate malnutrition	1.19	1.17	1.22	< 0.001
Mild malnutrition	1.17	1.15	1.19	< 0.001
Overweight	0.74	0.73	0.75	< 0.001
Obese	0.64	0.63	0.65	< 0.001
District				
Futian	1			
Baoan	2.00	1.97	2.04	0.888
Dapeng	3.23	3.10	3.36	< 0.001
Guangming	1.42	1.39	1.45	< 0.001
Longgang	1.81	1.78	1.83	< 0.001
Longhua	3.44	3.38	3.51	< 0.001
Luohu	2.53	2.48	2.50	< 0.001
Nanshan	1.68	1.65	1.71	< 0.001
Pingshan	1.12	1.10	1.16	< 0.001
Yantian	3.80	3.69	3.93	< 0.001
Sex				
Girl	1			
Boy	0.90	0.89	0.91	< 0.001
Age				
6–8	1			
9–11	0.62	0.61	0.62	< 0.001
12–14	0.23	0.22	0.23	< 0.001
15–17	0.21	0.21	0.22	< 0.001
≥ 18	0.26	0.25	0.28	< 0.001
Type of school				
Public	1			
Private	1.23	1.22	1.24	< 0.001

## Discussion

This large population-based study found that the average prevalence of dental caries was 41.15%; the mean dmft/DMFT score and the overall caries restoration rate were 1.24 and 10.32%, respectively. The prevalence of caries was higher in primary teeth. Caries was more frequently observed among girls. Disparities in oral health conditions were found across administrative regions and type of school. Moreover, an inverse association between caries and BMI was demonstrated. Children with malnutrition were more likely to develop dental caries than those with normal weight or high BMI.

Compared with the results from the Third National Oral Health Epidemiologic Survey in 2005 [20], prevalence of caries has decreased in the recent decade, indicating an achievement in managing oral health problems. However, the prevalence of caries in some age groups remained above the target of having more than half of children free from caries, recommended by WHO and Federation of Dentistry International [21].

The prevalence of caries in primary and permanent teeth were slightly lower than that obtained from the national survey in the same year (70.1 and 34.5%, respectively). One possible explanation is that samples in this study were collected only in Shenzhen city, which has a higher socioeconomic level and a better education standard than other cities.

Compared with the prevalence of dental caries in other developed [17] and developing [22–24] countries, the findings in this survey indicated a lower prevalence. On the other hand, a high prevalence of primary caries was observed in the study population, affecting more than half the participants younger than 10 years. In addition, the general caries restoration rates of primary and permanent teeth were significantly lower than the average level in developed countries. Caries restoration rate is the proportion of students who received cavity restoration treatment among students with diagnosed caries. The restoration rate of primary teeth among 6-year-old children in Japan was 84% [25]. More than 90% of children in Australia had their permanent teeth caries restorated [26]. Only 10.30% of students with caries received restorative treatment in our study population, which highlights the necessity of health education on encourage regular oral health check-ups and treatment among students in Shenzhen. Moreover, the high prevalence and low restorative rate of primary teeth partly reflects parents’ general attitude toward caries protection: Since the primary teeth will be replaced with the permanent teeth, it is not necessary to protect or restore primary teeth caries [27]. According to a previous study [28], caries and missing primary teeth are major causes of permanent teeth caries. Therefore, effective measures to improve the health of primary teeth such as education programs and free dental consultation clinics are warranted.

Sex differences in dental caries and restorative rates were identified in agreement with other studies in China [7, 29]. The following reasons may contribute to these association: firstly, girls are more fond of sweets and snacks [30]; secondly, the development of teeth regularly appeared earlier among girls [31].

Maps above show regional disparities in students’ oral health status. Districts located centrally (Futian, Luohu, Longgangand Nanshan) had a generally lower prevalence and higher restoration rates of dental caries than other areas. According to one recent report [32], the four districts mentioned above ranked high in relation to gross domestic product (GDP) increase in 2017. Since management of students’ health, including oral health, was administrated at district level in Shenzhen, a higher regional GDP increase was possibly associated with higher family socioeconomic levels and better government financial support.

Additionally, public school students in Shenzhen showed a higher oral health status in our study. Gerdin’s group reported a decrease in caries prevalence with better socioeconomic status in all age groups [33], which may explain the disparities across districts and type of school. Nevertheless, we used data collected from the Shenzhen students’ health surveillance system. Because detailed information on students’ family wealth was not applicable, we could not conclude that disparities between public and private schools were related to students’ socioeconomic status. Further study may be necessary to identify the socioeconomic factors associated with school disparities and to enhance awareness of dental health among children in private schools.

The increasing prevalence of obesity in children has been linked to the consumption of a sugar-rich diet, according to a longitudinal study [34], and diet was also associated with caries experience [13]. Common determinants such as poor diet’s increasing the risk of both caries and obesity indicated a potential link between those diseases. This association has been suggested in some studies [35–37], which reported that students with higher BMI were less likely to be caries free.

Here, we found that students in the lower BMI categories had higher risk of experiencing caries in both girls and boys. Similar results have been reported based on data from large surveys in Guangzhou [38] and the United States [39]. Liang et al. have demonstrated that overweight and obese children had lower odds for primary dental caries after adjusting for age and sex in a cross-sectional study in Guangzhou city [38]. Also, according to data from the National Health And Nutrition Examination Survey III in the United States [39], being overweight may be related to decreased rates of caries among children aged 2 to 18 years. However, a separate study (although with a smaller sample size of 835 participants) found no significant relationship between BMI and caries [13]. The significant disagreement among studies may be attributed to differences in the target population and caries severity. Severe caries are usually followed by toothache, which may reduce children’s ability and willingness to eat, and thereby reduce their food intake. In addition, methods of categorizing BMI were different in these studies [13, 39–42]. Some studies employed CDC centiles [39, 40], while others used cut-off values recommended by the International Obesity Task Force [13, 41] or WHO [42]. One study also used BMI as a continuous variable [13]. In our study, the threshold was recommended by the Group of China Obesity TaskForce [19].

Our study supported an inverse relationship between dental caries and BMI categories. One possible explanation is that because of the one child policy in China, children have become “little emperors” at home. Family members (parents and grandparents) have spent more money on food, and most of them still believed that higher food intake makes their children grow faster. At the same time, they paid more attention to their child’s oral health status. As a result, children with high BMI might have low odds of caries experience. However, this hypothesis could not be tested in our study since the information on parents’ knowledge and attitude to diet and oral health is not available, further study is required. Moreover, caries severity was not recorded in the present survey. It is possible that students with malnutrition were more likely to have severe caries compared with their peers. As a consequence of malnutrition, enamel hypoplasia, salivary glandular hypofunction, and saliva compositional changes [43] might be potential sequelae linked to malnutrition and caries. In return, severe dental caries might result in a reduced food intake and thereby malnutrition, as mentioned above. Further studies with a longitudinal study design and comprehensive collections of socioeconomic backgrounds would be helpful in understanding the casual relationship.

To the best of our knowledge, this is the first large population-based survey in Shenzhen. The spatial and school-related disparities in childhood oral health found in this study could be the basis for policy implementation in the future. However, some limitations also exist in this study. First, we did not collect the information on dietary habits and family socioeconomic status, which may bias the observed association between oral health indicators and overweight/obesity, although it should not affect the accuracy of findings regarding dental caries status. Second, considering that the sample included all the school-aged students at school and senior high school is not covered in compulsory education, more efforts are required to assess whether these findings children who chose to leave school after junior high school. Moreover, no causal association could be summarized as with other cross-sectional studies, further longitudinal studies and randomized controlled trials need to be conducted.

## Conclusion

We found a high prevalence of primary teeth caries, low caries restoration rates, and regional disparities in school children in Shenzhen. Children who were overweight or obese had a lower caries rate than their peers in the malnutrition groups. Our results provide valuable information for implementing measures to improve the dental health of school children. Further studies are required to identify more factors associated with oral health problems.

## Abbreviations

ANOVA: Analysis of variance
BMI: Body mass index
CDC: Center for disease control and prevention
CPI: Community periodontal index
DMFT: Decayed, missing, and filled teeth index for permanent teeth
dmft: Decayed, missing, and filled teeth index for primary teeth
GDP: Gross domestic product
WHO: World Health Organization

## Availability of data and materials

The data sets generated and analyzed during the current study are not publicly available because these data are stored in a government system, but are available from the corresponding author on reasonable request.
